# Assessment of intestinal barrier integrity and associations with innate immune activation and metabolic syndrome in acutely ill, antipsychotic-free schizophrenia patients

**DOI:** 10.1186/s12974-025-03584-3

**Published:** 2025-10-13

**Authors:** Kaushiki Mukherjee, Paul C. Guest, Madeleine Nussbaumer, Leon Dudeck, Leila Shokati Asl, Gabriela Meyer-Lotz, Henrik Dobrowolny, Katrin Borucki, Hans-Gert Bernstein, Alexander Link, Borna Relja, Kolja Schiltz, Thomas Nickl-Jockschat, Johann Steiner

**Affiliations:** 1 Department of Psychiatry and Psychotherapy, Otto-von-Guericke-University Magdeburg, Leipziger Str. 44, Magdeburg, 39120 Germany; 2 Laboratory of Translational Psychiatry, Otto-von-Guericke-University Magdeburg, Magdeburg, Germany; 3 Department of Biochemistry and Tissue Biology, Laboratory of Neuroproteomics, Institute of Biology, University of Campinas (UNICAMP), Campinas, Brazil; 4 Institute of Clinical Chemistry and Pathobiochemistry, Otto-von-Guericke-University Magdeburg, Magdeburg, Germany; 5 Department of Gastroenterology, Hepatology and Infectious Diseases, Otto-von-Guericke-University Magdeburg, Magdeburg, Germany; 6 Department of Trauma, Hand, Plastic and Reconstructive Surgery, Translational and Experimental Trauma Research, Ulm University Medical Center, Ulm, Germany; 7 Center for Behavioral Brain Sciences (CBBS), Magdeburg, Germany; 8 Department of Forensic Psychiatry, Psychiatric Hospital of the Ludwig-Maximilians-University, Munich, Germany; 9 Department of Psychiatry, Iowa Neuroscience Institute, Department of Neuroscience and Pharmacology, University of Iowa, Iowa City, USA; 10 German Center for Mental Health (DZPG), Partner Site Halle-Jena-Magdeburg, Magdeburg, Germany

**Keywords:** Schizophrenia, Gut–brain axis, Intestinal permeability, Lipopolysaccharide Binding protein (LBP), Intestinal fatty acid Binding protein (I-FABP), Innate immunity, C - Reactive protein (CRP), Metabolic syndrome, Leaky gut, Unmedicated psychosis

## Abstract

**Background:**

Schizophrenia (Sz), once seen solely as a brain disorder, is now recognised as a systemic illness involving immune and metabolic dysregulation. The intestinal barrier has emerged as a key player in gut–brain–immune interactions. However, studies in early, antipsychotic free stages remain scarce and often neglect confounding factors such as smoking and metabolic syndrome.

**Methods:**

We measured two complementary markers: lipopolysaccharide-binding protein (LBP), reflecting endotoxin exposure and systemic immune activation, and intestinal fatty acid-binding protein (I-FABP), indicating gut epithelial damage and permeability changes, in blood from 96 acutely ill, antipsychotic-free Sz patients (61 first-episode, 35 relapsed) and 96 matched controls. Associations with innate immunity, metabolic parameters, smoking, and clinical features were assessed using nonparametric statistics and random forest regression. Group differences were tested using covariate adjustment, as well as in a separate analysis of non-smokers (Sz: *n* = 42; controls: *n* = 84).

**Results:**

Median LBP was higher in Sz (21.96 µg/mL) vs. controls (18.10 µg/mL; FDR-adjusted *p* = 0.021, δ = 0.209) but became non-significant after adjusting for smoking (FDR-adjusted *p* = 0.199). In contrast, I-FABP was lower in Sz (218.2 pg/mL) than controls (315.0 pg/mL; FDR-adjusted *p* = 0.021, δ = –0.195) and remained robust across smoking-adjusted analyses. No differences were found between first-episode and relapsed patients for either marker.

LBP correlated strongly with CRP (*r* = 0.557, *p* < 0.001) and neutrophils (*r* = 0.468, *p* < 0.001) and was moderately predicted by immune models (pseudo-R^2^ = 0.354 overall; 0.273 Sz; 0.449 controls). Links to waist circumference and blood pressure were weaker (pseudo-R^2^: 0.048–0.104). I-FABP showed fewer immune associations and was not correlated with LBP (*r* = –0.017, FDR-adjusted *p* = 0.819), suggesting distinct mechanisms.

**Conclusions:**

Our findings suggest separable gut‑related processes in antipsychotic-free Sz. The apparent LBP elevation was not schizophrenia‑specific; its strong correlations with CRP and neutrophils point to smoking related inflammation rather than a schizophrenia specific effect. Accordingly, prior findings of LBP elevations in Sz likely reflect unaccounted smoking. In contrast, reduced I-FABP, independent of smoking, may indicate epithelial injury. The absent correlation between LBP and I-FABP highlights distinct pathophysiological dimensions of gut dysfunction. Longitudinal studies, ideally spanning prodromal phases and integrating microbiome, dietary, smoking, and permeability assessments, are needed to clarify temporal dynamics and guide stratified treatments.

**Graphical Abstract:**

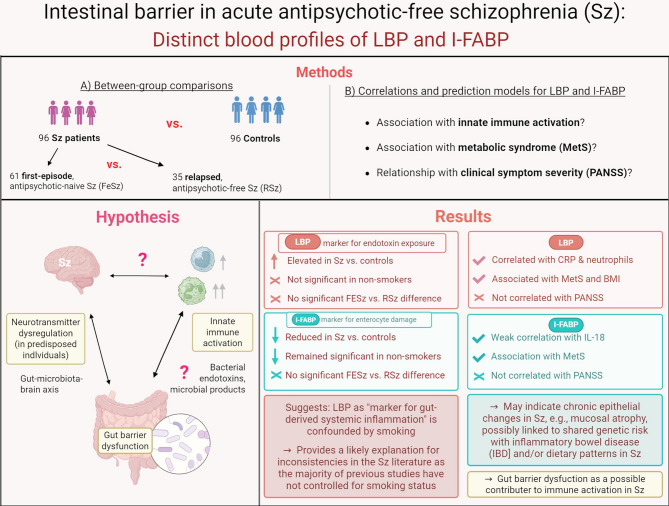

**Supplementary Information:**

The online version contains supplementary material available at 10.1186/s12974-025-03584-3.

## Background

Schizophrenia (Sz), once viewed solely as a brain disorder, is now recognized as a systemic illness with immune and metabolic components [1–3]. Meta-analyses have reported increased neutrophil and monocyte counts and slightly elevated C-reactive protein (CRP) in Sz [4, 5]. In our previous study of 253 acutely ill, unmedicated patients and 294 controls – excluding individuals with infections, trauma, or recent surgery – we found large and medium effect size elevations in neutrophils and monocytes, respectively, even after adjusting for stress and smoking [6]. This “sterile” myeloid activation raised the question of its underlying drivers.

One possible upstream mechanism is subclinical gut barrier dysfunction, which may allow translocation of microbial products such as lipopolysaccharide (LPS) into the circulation, triggering systemic inflammation. Supporting this, Gokulakrishnan et al. [7] found elevated gut permeability markers, including lipopolysaccharide-binding protein (LBP) and zonulin, especially in antipsychotic-naïve patients, suggesting intestinal barrier changes are intrinsic rather than drug-induced. Similarly, Weber et al. [8] reported elevated soluble CD14 (sCD14), a marker of monocyte activation, before Sz onset in U.S. military personnel, consistent with low-grade bacterial translocation and innate immune priming. LPS, a potent activator of neutrophils and monocytes [9–12], can stimulate cytokines such as interleukin (IL)−6, which drives hepatic CRP synthesis [13].

Although human data remain scarce, animal models suggest that gut-derived inflammation can impair neurodevelopment and behavior relevant to Sz phenotypes, including social cognition [14, 15]. These effects are mediated via the gut–microbiota–brain axis, encompassing immune signaling, the vagus nerve, neuroendocrine pathways, and microbial metabolites that influence brain function and behavior [15, 16]. Consistent with this, Sz patients exhibit gut microbiota dysbiosis alongside immune-inflammatory abnormalities [17, 18].

Increased intestinal permeability may also contribute to development of *metabolic syndrome (MetS)*^1^ by enabling endotoxin-driven low-grade inflammation [19–21], which impairs insulin signaling and promotes insulin resistance [22, 23]. While second-generation antipsychotics, particularly clozapine and olanzapine, contribute to weight gain and metabolic dysfunction [24, 25], such changes also occur in antipsychotic-naïve first-episode Sz (FESz) patients, and first-degree relatives show increased MetS risk [26–31], suggesting intrinsic immunometabolic vulnerability. These findings suggest that immunometabolic dysfunction may precede medication effects and could reflect intrinsic vulnerability. A bidirectional relationship between MetS and gut permeability has been proposed, in which systemic inflammation and oxidative stress disrupt the mucosa, creating a self-reinforcing cycle [32–35]. This link is particularly relevant in Sz, where both systemic inflammation and metabolic risk are elevated, even in early stages of the illness.

Studies on gut permeability markers in Sz have yielded mixed results. Some reported elevated LBP, intestinal fatty acid-binding protein (I-FABP), or zonulin [7, 36, 37], while others find no LBP differences [8, 38, 39] (Supplementary Table 1). LBP correlated with CRP [8, 39], IL-6 and tumour necrosis factor (TNF)-α [37], sCD14, [8, 39] and with body mass index (BMI) in some cohorts [37, 39]. However, leaky gut-MetS links have not been systematically studied. Variation across studies may reflect differences in medication status, disease stage, biomarker selection, control of lifestyle factors, and geographic microbiome differences [7, 8, 37, 39], highlighting the need for standardized protocols and antipsychotic-free samples.

To address these gaps, we examined gut permeability markers and their immune–metabolic relationships in unmedicated acutely ill Sz patients, including both FESz and relapsed Sz (RSz) cases, to determine whether barrier dysfunction is an early, trait-like feature or develops later in the illness course. We focused on LBP, a liver-derived marker of endotoxin exposure and systemic immune activation [40], and I-FABP, an enterocyte-derived marker of gut epithelial injury [41], as they are frequently used in Sz research and distinguish endotoxin-related inflammation from enterocyte stress [7, 8, 36–39].

We aimed to (1) compare LBP and I-FABP levels between Sz patients and controls, and between FESz and RSz patients; (2) examine associations with innate immune markers; (3) assess links to MetS features; and (4) evaluate relationships with clinical symptom severity. This multi-dimensional approach sought to clarify the role of gut barrier integrity in Sz immunometabolic and clinical profiles, independent of medication and illness chronicity.

## Methods

### Patients and controls

The study complied with German law, the Declaration of Helsinki, and was approved by the Institutional Review Board (approval number 110/07). All participants gave written informed consent. Acutely ill inpatients with Sz (*n* = 96; Table 1), were recruited at the Department of Psychiatry, University of Magdeburg, and diagnosed per ICD-10.

**Table 1:** Comparison of Sz patients with healthy controls

**Variables**	**Sz** median (Q1,Q3,n)	**Controls** median (Q1,Q3,n)	**Test**	**Test value**	***p*-value** **(FDR)**	**Effect size** (Cliff’s delta)
Demographic data & severity of clinical symptoms						
Age (years)	33.0 (26.3;44.5;96)	34.5 (27.0;45.8;96)	U-Test	W = 4471.5	0.724 (0.965)	−0.030
Sex (female/male)	m: 56/f: 40	m: 56/f: 40	χ²-Test	χ² = 0.0	1.000 (1.000)	0.000
BMI (kg/m^2^)	23.56 (20.69;27.13;96)	23.69 (21.78;27.57;96)	U-Test	W = 4426.5	0.638 (0.965)	−0.039
Tobacco smoking (yes/no)	yes: 54/no: 42	yes: 12/no: 84	χ²-Test	χ² = 38.811	** < 0.001 (< 0.001)**	0.461
Duration of illness (years)	0.0 (0.0;3.3;94)	-	-	-	-	-
PANSS total corr. (score)	35.00 (28.00;48.75;96)	-	-	-	-	-
PANSS-P corr. (score)	12.00 (9.00;16.00;96)	-	-	-	-	-
PANSS-N corr. (score)	7.00 (4.00;13.00;96)	-	-	-	-	-
PANSS-G corr. (score)	15.00 (12.00;20.00;96)	-	-	-	-	-
Leaky gut markers						
Plasma LBP (µg/mL)	21.96 (16.66;29.60;95)	18.10 (13.89;23.48;95)	U-Test	W = 5457.0	**0.013 (0.021)**	0.209
Serum I-FABP(pg/mL)	218.2 (116.4;369.9;93)	315.0 (174.5;533.5;94)	U-Test	W = 3518.5	**0.021 (0.021)**	−0.195
Immune-related parameters						
Neutrophils (× 10⁹/L)	4.81 (3.53;6.48;96)	3.01 (2.36;3.86;94)	U-Test	W = 7258.0	** < 0.001 (< 0.001)**	0.609
Monocytes (× 10⁹/L)	0.56 (0.43;0.73;96)	0.42 (0.32;0.60;94)	U-Test	W = 6158.0	** < 0.001 (< 0.001)**	0.365
CRP (mg/L)	1.35 (0.60;3.40;95)	0.90 (0.60;1.70;95)	U-Test	W = 5371.0	**0.023** (0.054**)**	0.190
IL-18 (pg/mL)	214.6 (172.3;300.3;94)	208.3 (159.4;274.2;96)	U-Test	W = 5072.0	0.140 (0.245)	0.124
IL-6 (pg/mL)	35.03 (22.68;52.99;84)	30.25 (18.00;67.19;90)	U-Test	W = 4033.5	0.446 (0.446)	0.067
TNF-α (pg/mL)	17.05 (11.04;26.97;87)	20.50 (10.84;35.62;91)	U-Test	W = 3581.5	0.273 (0.319)	−0.095
MCP-1 (pg/mL)	277.4 (216.7;372.6;94)	308.5 (221.2;407.0;95)	U-Test	W = 4026.0	0.244 (0.319)	−0.098
Metabolic syndrome-related parameters						
MetS (yes/no)	yes: 10/no: 86	yes: 12/no: 84	χ²-Test	χ² = 0.051	0.821 (0.902)	0.033
Waist circumference (cm)	88.00 (80.50;97.25;94)	90.00 (80.25;97.00;96)	U-Test	W = 4465.0	0.902 (0.902)	−0.010
Systolic blood pressure (mmHg)	125.5 (115.0;140.0;96)	120.0 (110.0;127.5;96)	U-Test	W = 5799.0	**0.002 (0.015)**	0.258
Diastolic blood pressure (mmHg)	80.0 (70.0;90.0;96)	80.0 (70.0;80.0;96)	U-Test	W = 4491.0	0.754 (0.902)	−0.025
Triglycerides (mmol/L)	0.89 (0.64;1.21;96)	1.015 (0.695;1.427;96)	U-Test	W = 3974.5	0.100 (0.225)	−0.137
HDL cholesterol (mmol/L)	1.43 (1.20;1.78;96)	1.54 (1.25;1.79;96)	U-Test	W = 4073.5	0.165 (0.298)	−0.116
Glucose (mmol/L)	4.91 (4.49;5.53;94)	5.01 (4.71;5.30;94)	U-Test	W = 4212.0	0.582 (0.873)	−0.047
sRAGE (pg/mL)	1349 (785;1946;95)	1538 (1042;2547;96)	U-Test	W = 3667.0	**0.019** (0.059)	−0.196
VEGF (pg/mL)	232.5 (155.2;325.7;92)	175.7 (139.9;262.1;96)	U-Test	W = 5287.0	**0.020** (0.059)	0.197

Demographic data, clinical assessments, LBP and I-FABP measurements, and immune- and metabolic syndrome-related parameters

Data are presented as median (quartile 1; quartile 3; sample size) or number of cases. Significant p-values are highlighted in bold font

*Abbreviations: BMI* body mass index, *CRP* C-reactive protein, *FDR* false discovery rate-corrected *p*-value, *I-FABP* intestinal fatty acid binding protein, *IL-18* interleukin 18, *IL-6* interleukin 6, *LBP* lipopolysaccharide binding protein, *MCP-1* monocyte chemoattractant protein 1, *PANSS* positive and negative syndrome scale [PANSS scores were corrected (corr.) by subtraction of minimum scores, which represented no symptoms], *HDL* high density lipoprotein, *sRAGE* soluble receptor for advanced glycation end products, *TNF-* *α* tumor necrosis factor alpha, *VEGF* vascular endothelial growth factor

Exclusion criteria were psychosis secondary to medical conditions, infections (respiratory, gastrointestinal, urinary tract), substance use disorders, diabetes, immune diseases, cancer, or cardiovascular disease. Screening followed German AWMF schizophrenia guidelines and included physical examination, differential blood counts, CRP, kidney function, lipids, fasting glucose, urine drug screen, MRI, and EEG. Pathological results were defined using institutional reference ranges. Symptoms were rated with the Positive and Negative Syndrome Scale (PANSS).

Two patient subgroups were analyzed (Suppl. Table 2): (1) FESz (*n* = 61) naïve to antipsychotics at baseline (T0), and (2) RSz (*n* = 35) patients with longer illness duration(median 6.0 years) but antipsychotic-free at least 6 weeks before sample collection. Healthy controls (*n* = 96), matched for age, sex, and smoking status (Table 1), were recruited from the community and underwent identical screening. Controls with psychiatric illness, substance use, infections, diabetes, immune disorders, cancer, or cardiovascular disease were excluded.

### Blood samples

Overnight-fasted samples were collected within 24 h of admission (8:00 a.m.). Differential white blood cell (WBC) counts were obtained from EDTA tubes within one hour after blood take collection. Serum tubes were clotted for two hours, then centrifuged at 1000 g for 10 min; plasma EDTA tubes were centrifuged immediately. Supernatants were aliquoted and stored at − 80 °C until analysis.

### Assays

Serum I-FABP and plasma LBP were measured using commercial ELISAs (Hycult Biotech, Uden, Netherlands). Neutrophil and monocyte counts were obtained with an XN-3000 counter (Sysmex). CRP was measured on a Cobas 8000 c701 analyzer (Roche Diagnostics). IL-18, IL-6, TNF-α, MCP-1, soluble receptor for advanced glycation end products (sRAGE), and vascular endothelial growth factor (VEGF) were quantified with the LegendPlex Human Neuroinflammation Panel (BioLegend, San Diego, CA, USA).

### Statistics

Analyses were performed in R 4.3.1, with *p* < 0.05 (two-tailed) considered significant. False discovery rate (FDR) correction was applied within each variable domain using the method of Benjamini-Hochberg (Table 1). Data distributions were assessed by Shapiro–Wilk tests; non-parametric tests were used as most variables were non-normal.

#### Group comparisons

Demographic differences in sex and smoking status were assessed using χ²-square tests. Group comparisons for continuous variables (e.g., LBP and I-FABP) were conducted using non-parametric Mann–Whitney U-tests, and included comparisons between Sz patients and controls, FESz and RSz patients, and MetS status (MetS was defined as having three or more of the following risk factors for heart disease, stroke, or type 2 diabetes: abdominal obesity, high blood pressure, high triglycerides, low HDL cholesterol, and high blood sugar). Additional comparisons of LBP and I-FABP levels by sex and smoking status were performed using Mann–Whitney U-tests. Cliff’s delta (δ) was used to assess effect sizes (ǀδǀ ≥ 0.147 “small,” ǀδǀ ≥ 0.330 “medium,” ǀδǀ ≥ 0.474 “large”).

#### Correlation analyses

We computed Spearman’s rank correlation coefficients between LBP/I-FABP and (a) age, BMI; (b) duration of illness, PANSS scores; (c) innate immune markers; (d) MetS-related parameters (waist circumference, blood pressure, triglycerides, HDL cholesterol, glucose, sRAGE, VEGF).^2^ Analyses were run in the full sample and separately in Sz and controls.

#### Exploratory prediction models

To complement the correlation analyses, we conducted random forest regression with backward variable selection in the full sample, the Sz and control groups to identify the most robust predictors of LBP and I-FABP levels. Case weights adjusted for unequal observation counts (e.g., repeated measures or varying subject contributions). Model selection was based on highest pseudo-R^2^ (interpreted as: < 0.20 negligible/weak, 0.20–0.40 moderate, > 0.40 strong predictive power).

#### Smoking adjustment

Group differences in LBP and I-FABP were retested via Aligned Rank Transform (ART) ANOVA with smoking as covariate. A subgroup analysis of non-smokers used Mann–Whitney U-tests.

## Results

### Sensitivity and validity of I-FABP and LBP blood measures

Of 192 samples, 190 were within the detection range for LBP (2 below limit); 187 were within range for I-FABP (5 below limit).

### Group comparisons of demographic data and severity of clinical symptoms

Patients and controls were matched for age, sex, and BMI (Table 1, Supplementary Table 2). Smoking was more frequent in patients (*p* < 0.001; Table 1). As expected, FESz patients had shorter illness durations than RSz patients (*p* < 0.001), while PANSS total scores were similar, with a nominally higher PANSS-G score in FESz (*p* = 0.031; not significant after FDR correction) (Supplementary Table 2).

### Group comparisons in LBP and I-FABP levels

Median LBP was higher in Sz (21.96 µg/mL) vs. controls (18.10 µg/mL; *p* = 0.013, FDR-adjusted = 0.021, δ = 0.209), while I-FABP was lower (218.2 vs. 315.0 pg/mL; *p* = 0.021, FDR-adjusted = 0.021, δ = − 0.195; Table 1). No differences were found between FESz and RSz (Supplementary Table 2).

MetS status did not significantly affect LBP or I-FABP in patients (Supplementary Table 3). In controls, LBP was higher with MetS (25.78 vs. 17.24 µg/mL; FDR-adjusted < 0.001, δ = 0.610; Supplementary Table 4).

### Group comparisons in immune- and metabolic syndrome-related parameters

Compared to controls, Sz patients had elevated neutrophils (FDR < 0.001, δ = 0.609) and monocytes (FDR-adjusted < 0.001, δ = 0.365), with CRP showing a trend level difference (FDR-adjusted = 0.054, δ = 0.190). No group differences were seen for IL-18, IL-6, TNF-α, or MCP-1, nor between FESz and RSz (Supplementary Table 2).

MetS prevalence was similar between groups (Table 1). Waist circumference, diastolic blood pressure, triglycerides, HDL, and glucose did not differ, but systolic blood pressure was higher in patients (FDR-adjusted = 0.015, δ = 0.258). VEGF and sRAGE showed trend-level differences (FDR-adjusted = 0.059 each). No metabolic differences were found between FESz and RSz (Supplementary Table 2).

### Correlations and prediction models for LBP and I-FABP

To examine links between LBP/I-FABP and innate immune, metabolic, demographic, and clinical parameters, we used Spearman’s correlations (including Mann–Whitney U-tests for sex and smoking) and random forest regression in the full sample, and separately in Sz and control groups (Supplementary Tables 5 and 6).

#### LBP findings

LBP was significantly associated with most immune markers. CRP and neutrophil count were top predictors in all immune-based models, explaining a moderate proportion of variance (pseudo-R^2^: 0.354 full sample; 0.273 Sz; 0.449 controls), consistent with strong correlations (all FDR-adjusted < 0.001). Monocytes and IL-18 correlated in the full sample and/or controls, but contributed less to prediction. IL-6, TNF-α, and MCP-1 showed no significant correlations and low importance.

*MetS-*associations were weaker. Waist circumference and systolic blood pressure were the most relevant predictors, with waist circumference consistently correlated with LBP (FDR-adjusted < 0.001 full sample; *p* = 0.017 Sz/controls) and high importance in models. Predictive power was negligible to low (pseudo-R^2^: 0.048–0.104).

LBP correlated with BMI (*r* = 0.304, *p* < 0.001) and smoking (*p* = 0.005) in the full sample; BMI was a top-ranked predictor but with low utility (pseudo-R^2^: 0.097 full; 0.139 controls; –0.011 Sz). Age and sex were not associated. PANSS scores and illness duration were unrelated to LBP and explained no variance in the Sz model (pseudo-*R*^2^ = –0.279).

#### I-FABP findings

Associations with innate immunity were weaker than for LBP. IL-18 was the only immune marker significantly correlated with I-FABP—in the full sample (*r* = 0.199, FDR-adjusted = 0.046) and more strongly in Sz (*r* = 0.336, FDR-adjusted = 0.008). In models, IL-18 predicted I-FABP in Sz (pseudo-*R*^2^ = 0.167) but did not account significantly for variance in the full sample (0.024) or controls (–0.023). Monocytes and IL-6 showed moderate to high importance in Sz, but there were no significant correlations.

Metabolic predictors did not show significant correlations with I-FABP in any group and prediction models had negligible explanatory power (pseudo-R^2^ range: −0.117 to 0.015).

No associations with BMI, age, sex, or smoking. Models performed poorly (pseudo-R^2^: 0.015 full; –0.061 Sz; –0.042 controls). Clinical parameters were also unrelated to I-FABP, with negative pseudo-R^2^ in Sz (–0.132).

#### Correlation between LBP and I-FABP

Across all groups, no significant correlations were found between LBP and I-FABP levels. In the full sample, the correlation was near zero (*r* = –0.017, FDR-adjusted *p* = 0.819), with similarly negligible and non-significant correlations observed in controls, all Sz patients, FESz, and RSz subgroups (all FDR-adjusted *p*-values > 0.8; Supplementary Table 7).

### Testing robustness of LBP and I-FABP findings

#### Group differences adjusted for smoking and non-smoker subgroup analyses

After adjusting for smoking via ART ANOVA, LBP group differences were no longer significant (FDR-adjusted = 0.199), while I-FABP remained significant (FDR-adjusted = 0.033).

Similarly, focusing on the subgroup of non-smoking participants, LBP differences were non-significant (FDR-adjusted = 0.211), but I-FABP remained lower in patients (FDR-adjusted = 0.003, δ = − 0.350; Supplementary Table 8).

#### Correlation and prediction models for LBP in non-smokers

In non-smokers (42 Sz, 84 controls), CRP remained the strongest LBP predictor (pseudo-R^2^: 0.347 Sz; 0.385 controls; Supplementary Table 9). Neutrophils remained significant but less predictive; monocyte associations weakened.

Regarding metabolic predictors, waist circumference correlated with LBP in Sz (*r* = 0.448, *p* = 0.027) and was consistently a high-importance predictor. Systolic BP was a low-importance, but significant, predictor in Sz (*p* = 0.034). Cholesterol correlated with LBP only in controls (*r* = 0.333, *p* = 0.016).

## Discussion

This is the first study to examine LBP and I-FABP as gut barrier markers in a well-characterised cohort of acutely ill, antipsychotic-free Sz patients, and to examine their relationships with innate immune activation, metabolic features, and clinical characteristics. Our findings revealed a robust reduction in I-FABP levels in patients compared to controls, independently of smoking. However, the initially observed elevation in LBP was no longer present after accounting for smoking. These results point to a selective alteration in gut barrier function in Sz, specifically affecting enterocyte integrity as reflected by the I-FABP marker, rather than endotoxin exposure as measured by LBP.

Importantly, although LBP showed moderate correlations with immune markers such as CRP and neutrophils, these relationships also appear to be driven or amplified by smoking, which was prevalent among patients. The small effect sizes for LBP and I-FABP contrast with the larger elevations in innate immune cells, suggesting that gut barrier changes, while detectable, may not be the primary driver of immune activation during acute psychosis. However, we cannot rule out the possibility that earlier, transient disruptions in gut integrity may have contributed to the immune profile seen at presentation. Longitudinal studies, ideally including ultra-high-risk and prodromal populations, are needed to determine whether gut barrier dysfunction precedes or follows immune activation in Sz.

### LBP: Links to endotoxin burden and innate immune activation

Our findings align with prior reports of elevated LBP in Sz [7, 36, 37] (Supplementary Table 1), and its consistent correlations with CRP and neutrophils support its role as a gut-derived inflammation marker [40]. In contrast, Severance et al. [39] and Scheurink et al. [38] have observed no increase in LBP (Supplementary Table 1). These discrepancies likely reflect differences in study populations and unaddressed confounders – for example, variability in illness stage (first-episode vs. chronic patients), prior medication exposure, and lifestyle factors. Importantly, smoking emerged as a critical factor, as the initially higher LBP in our antipsychotic-free patient sample was entirely attributable to smoking. Once smoking status was accounted for, the case–control difference in LBP disappeared. Given that many previous studies did not control for smoking, any observed LBP elevations may have been spurious, driven by higher smoking rates in patient groups rather than by schizophrenia itself. This finding offers a unifying explanation for prior inconsistencies and underscores the importance of controlling for smoking when evaluating LBP as an indicator of gut barrier dysfunction in schizophrenia.

Smoking may increase LBP through multiple mechanisms, including pulmonary exposure to bacterial endotoxins [42, 43] and increased disruption of gut barrier integrity [44]. Given the high prevalence of smoking in Sz populations, these pathways could confound attempts to interpret LBP as a gut-specific marker of barrier dysfunction.

This suggests that LBP reflects low-grade endotoxin burden or immune system activation in a subset of patients. However, these associations must be interpreted with caution, as they may reflect shared variance with smoking or other lifestyle-related factors. Taken together, these findings highlight the importance of controlling for smoking when using LBP as a biomarker of gut permeability, and suggest that LBP is not a reliable indicator of gut-derived immune activation in Sz without proper stratification. These findings may help to clarify the inconsistencies observed in earlier research.

### I-FABP: Links to enterocyte integrity and MetS

I-FABP, reflecting enterocyte injury [41, 45], was reduced in Sz and unaffected by smoking, suggesting a more stable alteration. This pattern may indicate chronic epithelial changes, e.g., mucosal atrophy from long-standing low-grade inflammation, possibly linked to shared genetic risk with inflammatory bowel disease (IBD)^3^ and/or dietary patterns in Sz such as a preference for sweet and high-fat foods [46–49]. Unlike our results, Jensen et al. [36] found elevated I-FABP in medicated chronic Sz, and González-Blanco et al. [37] found no differences. These discrepancies could reflect differences in disease phase, antipsychotic exposure, and/or methodological designs (Supplementary Table 1).

### Divergent gut marker profiles and implications for pathophysiology

The opposite patterns of elevated LBP and reduced I-FABP indicate that gut barrier dysfunction in Sz may reflect distinct processes such as paracellular leakiness and chronic enterocyte dysfunction, respectively [45, 50]. Their lack of correlation supports this distinction. Only LBP was consistently linked to inflammation, emphasizing the importance of using multiple markers rather than single marker approaches in capturing gut barrier complexity.

The moderate association of LBP with waist circumference and systolic BP fit with evidence that visceral adiposity and metabolic stress impair gut integrity [51–53]. Emerging evidence supports a *bidirectional relationship* between MetS and gut barrier integrity [51, 53–55].

Metabolic disturbances like hyperglycemia, which may play a role in Sz [28, 29], can compromise gut integrity. Elevated glucose levels disrupt tight junctions through GLUT2-mediated metabolic reprogramming in intestinal epithelial cells, increasing paracellular permeability [56, 57]. Moreover, systemic inflammation driven by MetS-associated cytokines such as TNF-α and IL-6 can downregulate junctional proteins and impair epithelial structure [58–60]. Dyslipidemia and oxidative stress may further damage enterocytes, potentially contributing to reduced I-FABP levels [52–54].

In the reverse direction, increased gut permeability facilitates translocation of bacterial products like LPS, which activate innate immune responses and promote systemic inflammation. This, in turn, can exacerbate metabolic dysregulation and perpetuate intestinal barrier dysfunction, creating a self-reinforcing inflammatory-metabolic loop [51, 55, 61].

### Strengths and limitations

Key strengths include a systematically recruited, antipsychotic-free cohort, matched controls, and a dual-marker strategy distinguishing endotoxin-related inflammation from enterocyte stress (Supplementary Table 1). By employing a dual-marker approach, we were able to differentiate between distinct dimensions of gut dysfunction. Moreover, combining traditional correlation analyses with machine learning-based random forest models enhanced the robustness and interpretability of our findings.

Limitations of the study include the cross-sectional design, which precluded causal inference. We cannot determine whether gut barrier changes preceded or followed immune alterations. Furthermore, we lacked direct permeability testing (e.g., lactulose/mannitol or zonulin), dietary and microbiome data. Additionally, smoking differences between Sz and controls limit the interpretation of LBP findings.

## Conclusions and future directions

We identified two dissociable gut-barrier alterations in Sz: smoking-sensitive LBP elevations linked to systemic inflammation and smoking-independent I-FABP reductions. I-FABP may be a comparatively stable marker of gut epithelial status.

Future studies should determine whether these changes are state-dependent or trait-like by tracking individuals from pre-psychotic through acute and remitted phases and, critically, by assessing unaffected first-degree relatives to evaluate LBP and I-FABP as potential endophenotypes and indicators of familial vulnerability. Longitudinal designs that integrate gut-barrier markers with microbiome and dietary assessments, functional permeability testing, and targeted interventions (e.g., probiotics, anti-inflammatory agents, dietary modification) are needed to clarify mechanisms and pave the way toward more personalized treatments for Sz.

## Supplementary Information

Supplementary Material 1.

Supplementary Material 2.

## Data Availability

Online supplementary material.

## Abbreviations

ART: Aligned Rank Transform
AWMF: Association of the Scientific Medical Societies in Germany (Arbeitsgemeinschaft der Wissenschaftlichen Medizinischen Fachgesellschaften)
BMI: Body Mass Index
CBBS: Center for Behavioral Brain Sciences
CRP: C-Reactive Protein
DZPG: German Center for Mental Health (Deutsches Zentrum für Psychische Gesundheit)
EDTA: Ethylenediaminetetraacetic acid
ELISA: Enzyme-Linked Immunosorbent Assay
FDR: False Discovery Rate
FESz: First-Episode Schizophrenia
HDL: High-Density Lipoprotein
I-FABP: Intestinal Fatty Acid-Binding Protein
IL: Interleukin
LBP: Lipopolysaccharide-Binding Protein
LPS: Lipopolysaccharide
MCP-1: Monocyte Chemoattractant Protein-1
MetS: Metabolic Syndrome
PANSS: Positive and Negative Syndrome Scale
pg/mL: Picograms per Milliliter
R^2^: Coefficient of Determination
RSz: Relapsed Schizophrenia
sCD14: Soluble Cluster of Differentiation 14
sRAGE: Soluble Receptor for Advanced Glycation End Products
Sz: Schizophrenia
TNF-α: Tumor Necrosis Factor Alpha
VEGF: Vascular Endothelial Growth Factor
WBC: White Blood Cell

## Footnotes

1 The most widely accepted and best established criteria for metabolic syndrome are the National Cholesterol Education Program (NCEP) Adult Treatment Panel III (ATP III) criteria, which were updated by the American Heart Association (AHA). According to these, metabolic syndrome is diagnosed when an individual exhibits at least three of the following five risk factors: 1.) increased waist circumference (≥ 102 cm in men, ≥ 88 cm in women); 2.) elevated triglycerides (≥ 150 mg/dL or receiving treatment for high triglycerides); 3.) reduced HDL cholesterol (< 40 mg/dL in men, < 50 mg/dL in women, or undergoing treatment for low HDL); 4.) elevated blood pressure (≥ 130/85 mm Hg or receiving antihypertensive treatment); 5.) elevated fasting glucose (≥ 100 mg/dL or under treatment for high glucose).

2 Although not part of the formal diagnostic criteria for Metabolic Syndrome (MetS), sRAGE and VEGF were included as exploratory markers due to their mechanistic relevance to vascular and metabolic dysregulation. Soluble RAGE (sRAGE), a decoy receptor for advanced glycation end-products (AGEs), is inversely associated with insulin resistance, glucose intolerance, and type 2 diabetes [Yamagishi & Matsui (2016): https://doi.org/10.2741/e178; Geroldi et al. (2005): https://doi.org/10.1097/01.hjh.0000177535.45785.64]. VEGF (vascular endothelial growth factor) regulates angiogenesis and endothelial function and has been linked to visceral adiposity, adipose tissue inflammation, and insulin resistance in obesity and MetS [Silha et al. (2005): https://doi.org/10.1038/sj.ijo.0802987].

3 In coeliac disease and Crohn’s disease, I-FABP levels increase during active mucosal injury, but may decrease in the chronic stage, correlating with the extent of enterocyte damage.
