# Supplementary material for: Assessment of intestinal barrier integrity and associations with innate immune activation and metabolic syndrome in acutely ill, antipsychotic-free schizophrenia patients
Source: J Neuroinflammation. 2025 Oct 13;22:232. doi: 10.1186/s12974-025-03584-3 (PMC12519603; doi:10.1186/s12974-025-03584-3)
Supplement: Supplementary file 1 — Supplementary Material 1. [file 12974_2025_3584_MOESM1_ESM.docx]

**Supplementary Table 1: Studies investigating LBP and I-FABP levels in schizophrenia compared to controls**

| **Ref.** | **Sample sizes (schizophrenia / controls)** | **Recruitment location** | **Disease phase / inpatient or outpatient** | **Antipsychotic status** | **Marker** | **Assay method / manufacturer** | **Detection range** | **Main results** |
| --- | --- | --- | --- | --- | --- | --- | --- | --- |
| Severance et al., 2013 [1] | (1) 141 / 78  (2) 116 / 0  78 drug-naïve first-episode + 38 medicated = 116 | (1) Sheppard Pratt Health System, Baltimore, USA  (2) University of Cologne, Cologne, Germany | (1) Chronic  (2) Mixed | (1) Medicated  (2) 78 Drug-naive / 38 medicated first episode schizophrenia | LBP  sCD14 | Cell Sciences Multispecies LBP ELISA  R&D Systems Human sCD14 Quantikine ELISA | 5ng/mL – 50 ng/mL  250 pg/mL- 16000 pg/mL |  **LBP**: No significant group difference between schizophrenia (9.4 ± 4.7 ng/mL)* and controls (8.2 ± 4.4 ng/mL)*  LBP correlated with CRP and BMI in schizophrenia.  No difference between drug-naïve and medicated schizophrenia patients. No effect of smoking status.   **sCD14**: Significantly higher levels in schizophrenia compared to controls (P<0.001). sCD14 correlated with CRP in schizophrenia but not with BMI. No significant effect of antipsychotic status on sCD14 levels. |
| Weber et al., 2018 [2] | 80 pre-onset / 80 | Multiple Military U.S. sites | Pre-onset | Drug-naive | LBP  sCD14 | Cell Sciences Multispecies LBP ELISA  R&D Systems Human sCD14 Quantikine ELISA | 5ng/mL – 50 ng/mL  250 pg/mL- 16000 pg/mL |  **LBP**: Samples from individuals who later developed schizophrenia exhibited a non-significant trend (P=0.06) toward lower LBP levels (17.76 ± 7.15 absorbance) than controls (19.97 ±8.16 absorbance). In males within this group, LBP levels correlated with CRP.   **sCD14**: Samples from individuals who later developed schizophrenia had significantly elevated sCD14 levels (P=0.02), suggesting that monocyte activation occurs prior to schizophrenia diagnosis.  sCD14 was not significantly correlated with CRP levels. |
| Gokulakrishnan et al., 2022 [3] | 40 / 20 | National Institute of Mental Health and Neurosciences (NIMHANS),  Bengaluru, India | Unclear / Unclear | 15 Antipsychotic-naïve  25 Medicated (Risperidone) | LBP  intestinal alkaline phosphatase (IAP)  Zonulin | Elabscience Human LBP ELISA  Cusabio Human IAP ELISA  Cusabio Human Zonulin ELISA | 0.78 ng/mL -50 ng/mL  3.12 ng/mL – 200 ng/mL  0.625 ng/mL – 40 ng/mL |  **LBP**: Significantly increased in schizophrenia (148.5 [110; 190] ng/mL)^#^ versus controls (97.7 [70; 115] ng/mL^#^; P<0.01). Higher levels of LBP in the antipsychotic-naïve subgroup.   **IAP**: Significantly reduced in schizophrenia patients (P<0.05), may indicate impaired intestinal detoxification capacity, potentially contributing to inflammation. Lower levels of IAP in the antipsychotic-naïve subgroup.   **Zonulin**: Significantly higher in schizophrenia (P<0.05), consistent with increased intestinal permeability, or "leaky gut". Higher zonulin levels in the antipsychotic-naïve subgroup. |
| Jensen et al., 2023 [4] | 389 / 418 | NORMENT Centre, Oslo, Norway | Subacute and Chronic / Mixed | Mixed | LBP  I-FABP | R&D systems Human LBP ELISA  R&D systems Human I-FABP ELISA | 0.8 ng/mL – 50 ng/mL  15.6 pg/mL – 1000 pg/mL |  **LBP**: Significantly higher in schizophrenia (27 [23;32] ng/mL) ^#^ versus controls (19 [16;23] ng/mL^#^; P< 0.05). Positive correlation between LBP and CRP. Negative association with antipsychotic use.   **I-FABP**: Significantly higher in schizophrenia (500 [470;520] pg/mL)^#^ versus controls (350 [320;370] pg/mL^#^; P< 0.001). |
| Scheurink et al., 2023 [5] | 72 / 39 | University of Utrecht, Utrecht, Netherlands | Unclear / In- and Outpatients | Unclear | LBP  sCD14 | R&D systems Human LBP ELISA  R&D systems Human sCD14 DuoSet ELISA | 0.8 ng/mL – 50 ng/mL  62.5 pg/mL – 4000 pg/mL |  **LBP**: No significant group difference between schizophrenia (8.26 ± 0.34 ng/mL) and controls (7.63 ± 0.39 ng/mL; P=0.25).   **sCD14**: No significant group difference between schizophrenia and controls (P=0.99).  Correlations or associations of LBP or sCD14 with CRP, BMI, antipsychotic medication or lifestyle factors such as smoking were not tested. |
| González-Blanco et al., 2024 [6] | 242 / 0 | Multicenter (4 centers), Spain | Chronic / Predominantly Outpatients | Medicated | LBP  LPS  I-FABP | RayBiotech Human LBP ELISA  Hycult Biotech LAL Chromogenic Endpoint Assay (LPS detection)  Cusabio I-FABP ELISA | 0.82 ng/mL – 200 ng/mL  0.04 EU/mL – 10 EU/mL  0.156 ng/mL – 10 ng/mL | Used reference values from the literature. LBP ≤15 μg/mL; LPS ≤0.5 U/mL; and I-FABP ≤2 ng/mL were considered as normal (no control sample):   **LBP**: Elevated in 62% of schizophrenia cases; associated with systemic inflammation. LBP correlated with BMI.   **LPS**: Elevated in 26% of schizophrenia cases.   **I-FABP**: Not significantly altered.   **LBP, LPS and I-FABP:** No significant correlation with the severity of psychotic symptoms or cognitive performance after controlling for confounding variables. |
| **Mukherjee et al. (this study)** | 95 / 96  60 drug-naïve + 35 antipsychotic-free for ≥6 weeks = 95 | Department of Psychiatry, University of Magdeburg, Magdeburg, Germany | Acute (60 first-episode, 35 relapsed) / Inpatients | 60 Antipsychotic-naïve  35 Antipsychotic--free for at least 6 weeks | LBP  I-FABP | Hycult Biotech Human LBP ELISA  Hycult Biotech Human I-FABP ELISA | 4.4 ng/mL – 50ng/mL  47 pg/mL – 3000 pg/mL |  **LBP**: Higher levels (P = 0.021) in schizophrenia (21.96 [16.66;29.6] ng/mL) versus controls 18.10 [13.89;23.48] ng/mL. Controlling for smoking made the results non-significant.  Significant correlations of LBP with CRP, neutrophil count, monocyte count and IL-18.   **I-FABP**: Significantly reduced in schizophrenia (218.2 [116.4;369.9] pg/mL) versus controls (315.0 [174.5;533.5] pg/mL; P=0.013). Not confounded by smoking. No correlation with CRP or differential white blood count.  **LBP** and **I-FABP** levels did not differ significantly between first-episode and relapsed patients. |

Annotation: *Means and standard errors of the mean (SEMs) were extracted from bar graphs; standard deviations (SDs) were then estimated by multiplying SEMs with the square root of the respective sample sizes. ^#^Medians and quartiles were extracted from box plots.

**References**

1. Severance EG, Gressitt KL, Stallings CR, Origoni AE, Khushalani S, Leweke FM, Dickerson FB, Yolken RH: **Discordant patterns of bacterial translocation markers and implications for innate immune imbalances in schizophrenia.** *Schizophr Res* 2013, **148:**130-137.

2. Weber NS, Gressitt KL, Cowan DN, Niebuhr DW, Yolken RH, Severance EG: **Monocyte activation detected prior to a diagnosis of schizophrenia in the US Military New Onset Psychosis Project (MNOPP).** *Schizophr Res* 2018, **197:**465-469.

3. Gokulakrishnan K, Nikhil J, Vs S, Holla B, Thirumoorthy C, Sandhya N, Nichenametla S, Pathak H, Shivakumar V, Debnath M, et al: **Altered Intestinal Permeability Biomarkers in Schizophrenia: A Possible Link with Subclinical Inflammation.** *Ann Neurosci* 2022, **29:**151-158.

4. Jensen SB, Sheikh MA, Akkouh IA, Szabo A, O'Connell KS, Lekva T, Engh JA, Agartz I, Elvsashagen T, Ormerod M, et al: **Elevated Systemic Levels of Markers Reflecting Intestinal Barrier Dysfunction and Inflammasome Activation Are Correlated in Severe Mental Illness.** *Schizophr Bull* 2023, **49:**635-645.

5. Scheurink TAW, Borkent J, Gangadin SS, El Aidy S, Mandl R, Sommer IEC: **Association between gut permeability, brain volume, and cognition in healthy participants and patients with schizophrenia spectrum disorder.** *Brain Behav* 2023, **13:**e3011.

6. Gonzalez-Blanco L, Dal Santo F, Garcia-Portilla MP, Alfonso M, Hernandez C, Sanchez-Autet M, Anmella G, Amoretti S, Safont G, Martin-Hernandez D, et al: **Intestinal permeability biomarkers in patients with schizophrenia: Additional support for the impact of lifestyle habits.** *Eur Psychiatry* 2024, **67:**e84.

**Supplementary Table 2:** **Comparison of first-episode (FESz) and relapsed (RSz) patients.** Demographic data, clinical assessments, LBP and I-FABP measurements, and immune- and metabolic syndrome-related parameters. Data are presented as median (quartile 1; quartile 3; sample size) or number of cases. Significant p-values are highlighted in bold font.

| **Variables** | **FESz** median (Q1,Q3,n) | **RSz** median (Q1,Q3,n) | **Test** | **Test value** | **p-value**  **(FDR)** | **Effect size** (Cliff's delta) |
| --- | --- | --- | --- | --- | --- | --- |
| ***Demographic data & severity of clinical symptoms*** | | | | | | |
| Age (years) | 31.0 (26.0;42.0;61) | 37.0 (27.0;48.0;35) | U-Test | W=896.5 | 0.194 (0.530) | -0.160 |
| Sex (female/male) | m: 33 / f: 28 | m: 23 / f: 12 | Χ²-Test | X²=0.803 | 0.370 (0.530) | 0.113 |
| BMI (kg/m^2^) | 23.74 (21.05;27.45;61) | 23.34 (20.32;26.79;35) | U-Test | W=1150.5 | 0.530 (0.530) | 0.078 |
| Tobacco smoking (yes/no) | yes: 32 / no: 29 | yes: 22 / no: 13 | Χ²-Test | X²=0.600 | 0.438 (0.530) | 0.101 |
| Duration of illness (years) | 0.0 (0.0;0.0;61) | 6.0 (2.0;10.0;33) | U-Test | W=61.0 | **<0.001 (<0.001)** | -0.939 |
| PANSS total corr. (score) | 36.00 (29.00;50.00;61) | 31.00 (24.00;42.00;35) | U-Test | W=1309.0 | 0.066 (0.101) | 0.226 |
| PANSS-P corr. (score) | 13.00 (9.00;15.50;61) | 12.00 (9.00;18.00;35) | U-Test | W=1051.5 | 0.906 (0.906) | -0.015 |
| PANSS-N corr. (score) | 8.00 (4.00;14.50;61) | 6.00 (2.00;11.00;35) | U-Test | W=1297.0 | 0.081 (0.101) | 0.215 |
| PANSS-G corr. (score) | 16.00 (12.50;22.00;61) | 13.00 (11.00;19.00;35) | U-Test | W=1350.5 | **0.031** (0.078) | 0.265 |
| ***Leaky gut markers*** | | | | | | |
| Plasma LBP (µg/mL) | 21.70 (16.12;30.21;61) | 22.19 (16.96;26.20;34) | U-Test | W=1009.0 | 0.831 (0.831) | -0.027 |
| Serum I-FABP(pg/mL) | 194.3 (80.9;376.8;59) | 245.1 (146.7;401.0;34) | U-Test | W=878.0 | 0.321 (0.641) | -0.125 |
| ***Immune-related parameters*** | | | | | | |
| Neutrophils (×10⁹/L) | 5.08 (3.59;6.58;61) | 4.51 (3.46;6.22;35) | U-Test | W=1176.0 | 0.411 (0.575) | 0.102 |
| Monocytes (×10⁹/L) | 0.60 (0.44;0.76;61) | 0.55 (0.41;0.66;35) | U-Test | W=1250.5 | 0.165 (0.384) | 0.171 |
| CRP (mg/L) | 1.26 (0.60;3.60;60) | 1.40 (0.60;3.30;35) | U-Test | W=976.5 | 0.571 (0.667) | -0.070 |
| IL-18 (pg/mL) | 214.9 (171.1;304.0;59) | 214.2 (172.7;290.7;35) | U-Test | W=1050.0 | 0.894 (0.894) | 0.017 |
| IL-6 (pg/mL) | 36.59 (22.69;57.64;51) | 31.02 (20.61;45.70;33) | U-Test | W=996.0 | 0.158 (0.384) | 0.184 |
| TNF-α (pg/mL) | 17.87 (13.34;28.18;53) | 16.29 (10.09;26.27;34) | U-Test | W=1067.0 | 0.150 (0.384) | 0.184 |
| MCP-1 (pg/mL) | 273.9 (214.3;371.8;59) | 323.3 (223.9;375.1;35) | U-Test | W=922.0 | 0.390 (0.575) | -0.107 |
| ***Metabolic syndrome-related parameters*** | | | | | | |
| MetS (yes/no) | yes: 7 / no: 54 | yes: 3 / no: 32 | Χ²-Test | X²=0.010 | 0.919 (0.981) | 0.046 |
| Waist circumference (cm) | 89.00 (79.00;97.00;59) | 87.00 (81.00;100.00;35) | U-Test | W=1036.0 | 0.981 (0.981) | 0.003 |
| Systolic blood pressure (mmHg) | 126.0 (120.0;140.0;61) | 120.0 (110.0;130.0;35) | U-Test | W=1208.5 | 0.278 (0.899) | 0.132 |
| Diastolic blood pressure (mmHg) | 80.0 (70.0;90.0;61) | 75.0 (70.0;80.0;35) | U-Test | W=1248.5 | 0.159 (0.899) | 0.170 |
| Triglycerides (mmol/L) | 0.88 (0.69;1.25;61) | 0.89 (0.62;1.18;35) | U-Test | W=1115.0 | 0.720 (0.981) | 0.044 |
| HDL cholesterol (mmol/L) | 1.39 (1.19;1.76;61) | 1.46 (1.22;1.87;35) | U-Test | W=958.5 | 0.409 (0.899) | -0.102 |
| Glucose (mmol/L) | 4.89 (4.50;5.55;60) | 5.01 (4.44;5.54;34) | U-Test | W=986.0 | 0.792 (0.981) | -0.033 |
| sRAGE (pg/mL) | 1390 (816;2055;60) | 1265 (683;1877;35) | U-Test | W=1138.0 | 0.500 (0.899) | 0.084 |
| VEGF (pg/mL) | 224.1 (146.2;317.1;58) | 250.2 (170.7;331.8;34) | U-Test | W=871.0 | 0.354 (0.899) | -0.117 |

*Abbreviations:* BMI=body mass index, CRP=C-reactive protein, FDR=false discover rate-corrected p-value, I-FABP=intestinal fatty acid binding protein, IL-18=interleukin 18, IL-6=interleukin 6, LBP=lipopolysaccharide binding protein, MCP-1=monocyte chemoattractant protein 1, PANSS=positive and negative syndrome scale [PANSS scores were corrected (corr.) by subtraction of minimum scores, which represented no symptoms], HDL=high density lipoprotein, sRAGE=soluble receptor for advanced glycation end products, TNF-a=tumor necrosis factor alpha, VEGF=vascular endothelial growth factor.

**Supplementary Table 3: Comparison of Sz patients with vs. without metabolic syndrome (MetS).** Data are presented as median (quartile 1; quartile 3; sample size) or number of cases. Significant p-values are highlighted in bold font.

| **Variables** | **Sz with MetS** median (Q1,Q3,n) | **Sz without MetS** median (Q1,Q3,n) | **Test** | **Test value** | **p-value**  **(FDR)** | **Effect size** (Cliff's delta) |
| --- | --- | --- | --- | --- | --- | --- |
| ***Demographic data & severity of clinical symptoms*** | | | | | | |
| Age (years) | 37.5 (29.0;47.8;10) | 32.5 (26.0;43.5;86) | U-Test | W=474.0 | 0.602 (0.651) | 0.102 |
| Sex (female/male) | m: 7 / f: 3 | m: 49 / f: 37 | Χ²-Test | X²=0.204 | 0.651 (0.651) | 0.081 |
| BMI (kg/m^2^) | 30.45 (26.43;35.48;10) | 23.48 (20.48;26.33;86) | U-Test | W=674.0 | **0.003 (0.014)** | 0.567 |
| Tobacco smoking (yes/no) | yes: 4 / no: 6 | yes: 50 / no: 36 | Χ²-Test | X²=0.574 | 0.449 (0.651) | 0.112 |
| Duration of illness (years) | 0.0 (0.0;9.3;10) | 0.0 (0.0;2.8;84) | U-Test | W=430.0 | 0.889 (0.889) | 0.024 |
| PANSS total corr. (score) | 39.00 (32.75;48.50;10) | 33.50 (27.75;49.00;86) | U-Test | W=507.0 | 0.359 (0.889) | 0.179 |
| PANSS-P corr. (score) | 15.00 (10.00;18.25;10) | 12.00 (8.00;16.00;86) | U-Test | W=533.0 | 0.218 (0.889) | 0.240 |
| PANSS-N corr. (score) | 6.50 (3.75;10.75;10) | 7.00 (4.00;13.00;86) | U-Test | W=414.5 | 0.857 (0.889) | -0.036 |
| PANSS-G corr. (score) | 15.50 (11.75;24.00;10) | 15.00 (11.75;20.00;86) | U-Test | W=474.5 | 0.597 (0.889) | 0.103 |
| ***Leaky gut markers*** | | | | | | |
| Plasma LBP (µg/mL) | 22.70 (18.28;32.67;10) | 21.22 (16.41;29.13;85) | U-Test | W=478.0 | 0.524 (0.524) | 0.125 |
| Serum I-FABP(pg/mL) | 430.3 (148.6;834.0;8) | 218.0 (115.1;357.2;85) | U-Test | W=451.0 | 0.130 (0.260) | 0.326 |
| ***Immune-related parameters*** | | | | | | |
| Neutrophils (×10⁹/L) | 5.72 (4.04;7.63;10) | 4.73 (3.50;6.35;86) | U-Test | W=517.0 | 0.300 (0.328) | 0.202 |
| Monocytes (×10⁹/L) | 0.64 (0.53;0.73;10) | 0.56 (0.43;0.73;86) | U-Test | W=512.0 | 0.328 (0.328) | 0.191 |
| CRP (mg/L) | 4.45 (0.90;6.78;10) | 1.21 (0.60;2.85;85) | U-Test | W=587.0 | **0.049** (0.321) | 0.381 |
| IL-18 (pg/mL) | 284.3 (194.3;326.7;10) | 212.9 (171.5;289.7;84) | U-Test | W=529.0 | 0.183 (0.321) | 0.260 |
| IL-6 (pg/mL) | 24.14 (19.62;39.63;7) | 35.47 (23.17;54.04;77) | U-Test | W=181.0 | 0.154 (0.321) | -0.328 |
| TNF-α (pg/mL) | 14.36 (12.00;15.95;9) | 17.82 (10.97;27.82;78) | U-Test | W=279.0 | 0.319 (0.328) | -0.205 |
| MCP-1 (pg/mL) | 374.0 (229.9;453.7;10) | 274.6 (214.3;363.7;84) | U-Test | W=553.0 | 0.104 (0.321) | 0.317 |
| ***Metabolic syndrome-related parameters*** | | | | | | |
| Waist circumference (cm) | 109.0 (100.5;114.2;10) | 87.50 (79.00;95.00;84) | U-Test | W=712.5 | **<0.001 (0.001)** | 0.696 |
| Systolic blood pressure (mmHg) | 140.0 (130.0;152.5;10) | 120.0 (115.0;130.0;86) | U-Test | W=700.0 | **0.001 (0.003)** | 0.628 |
| Diastolic blood pressure (mmHg) | 90.0 (77.5;100.0;10) | 80.0 (70.0;86.3;86) | U-Test | W=648.5 | **0.007 (0.015)** | 0.508 |
| Triglycerides (mmol/L) | 1.21 (0.94;3.20;10) | 0.88 (0.63;1.17;86) | U-Test | W=619.0 | **0.024 (0.038)** | 0.440 |
| HDL cholesterol (mmol/L) | 0.98 (0.84;1.19;10) | 1.45 (1.23;1.81;86) | U-Test | W=93.5 | **<0.001 (<0.001)** | -0.783 |
| Glucose (mmol/L) | 5.33 (4.50;6.13;10) | 4.87 (4.48;5.51;84) | U-Test | W=531.5 | 0.173 (0.198) | 0.265 |
| sRAGE (pg/mL) | 1081 (810;1757;10) | 1370 (723;2000;85) | U-Test | W=375.0 | 0.548 (0.548) | -0.118 |
| VEGF (pg/mL) | 183.9 (157.5;240.1;10) | 252.3 (153.9;330.3;82) | U-Test | W=279.0 | 0.102 (0.136) | -0.320 |

*Abbreviations:* BMI=body mass index, CRP=C-reactive protein, FDR=false discover rate-corrected p-value, I-FABP=intestinal fatty acid binding protein, IL-18=interleukin 18, IL-6=interleukin 6, LBP=lipopolysaccharide binding protein, MCP-1=monocyte chemoattractant protein 1, PANSS=positive and negative syndrome scale [PANSS scores were corrected (corr.) by subtraction of minimum scores, which represented no symptoms], HDL=high density lipoprotein, sRAGE=soluble receptor for advanced glycation end products, TNF-a=tumor necrosis factor alpha, VEGF=vascular endothelial growth factor.

**Supplementary Table 4: Comparison of controls with versus without MetS.** Data are presented as median (quartile 1; quartile 3; sample size) or number of cases. Significant p-values are highlighted in bold font.

| **Variables** | **Controls with MetS** median (Q1,Q3,n) | **Controls without MetS** median (Q1,Q3,n) | **Test** | **Test value** | **p-value**  **(FDR)** | **Effect size** (Cliff's delta) |
| --- | --- | --- | --- | --- | --- | --- |
| ***Demographic data & severity of clinical symptoms*** | | | | | | |
| Age (years) | 43.0 (33.5;49.0;12) | 32.0 (26.0;43.8;84) | U-Test | W=713.0 | **0.021 (0.042)** | 0.415 |
| Sex (female/male) | m: 8 / f: 4 | m: 48 / f: 36 | Χ²-Test | X²=0.098 | 0.754 (0.754) | 0.064 |
| BMI (kg/m^2^) | 30.17 (29.42;33.21;12) | 23.14 (21.67;25.85;84) | U-Test | W=891.0 | **<0.001 (<0.001)** | 0.768 |
| Tobacco smoking (yes/no) | yes: 0 / no: 12 | yes: 12 / no: 72 | Χ²-Test | X²=0.871 | 0.351 (0.468) | 0.143 |
| ***Leaky gut markers*** | | | | | | |
| Plasma LBP (µg/mL) | 25.78 (23.49;27.85;12) | 17.24 (13.77;22.12;83) | U-Test | W=802.0 | **<0.001 (<0.001)** | 0.610 |
| Serum I-FABP(pg/mL) | 295.3 (181.9;542.8;12) | 316.0 (173.8;533.5;82) | U-Test | W=484.5 | 0.937 (0.937) | -0.015 |
| ***Immune-related parameters*** | | | | | | |
| Neutrophils (×10⁹/L) | 3.80 (3.08;4.56;12) | 2.91 (2.35;3.76;82) | U-Test | W=668.0 | **0.047** (0.164) | 0.358 |
| Monocytes (×10⁹/L) | 0.53 (0.32;0.64;12) | 0.42 (0.32;0.59;82) | U-Test | W=570.0 | 0.380 (0.443) | 0.159 |
| CRP (mg/L) | 2.10 (1.08;3.35;12) | 0.80 (0.50;1.50;83) | U-Test | W=747.5 | **0.005 (0.036)** | 0.501 |
| IL-18 (pg/mL) | 253.2 (184.5;292.6;12) | 204.6 (156.5;267.6;84) | U-Test | W=643.0 | 0.125 (0.292) | 0.276 |
| IL-6 (pg/mL) | 24.62 (15.39;50.16;12) | 31.87 (18.46;69.74;78) | U-Test | W= 389.0 | 0.351 (0.443) | -0.169 |
| TNF-α (pg/mL) | 22.50 (8.88;33.56;12) | 19.54 (11.71;35.79;79) | U-Test | W=462.50 | 0.897 (0.897) | -0.024 |
| MCP-1 (pg/mL) | 339.6 (260.3;484.4;12) | 307.0 (216.3;392.2;83) | U-Test | W=585.0 | 0.333 (0.443) | 0.175 |
| ***Metabolic syndrome-related parameters*** | | | | | | |
| Waist circumference (cm) | 107.5 (98.0;113.8;12) | 86.50 (79.25;93.75;84) | U-Test | W=910.0 | **<0.001 (<0.001)** | 0.806 |
| Systolic blood pressure (mmHg) | 135.0 (120.0;141.5;12) | 120.0 (110.0;123.8;84) | U-Test | W=789.0 | **0.001 (0.002)** | 0.565 |
| Diastolic blood pressure (mmHg) | 90.0 (80.0;99.5;12) | 80.0 (70.0;80.0;84) | U-Test | W=812.0 | **<0.001 (<0.001)** | 0.611 |
| Triglycerides (mmol/L) | 2.03 (1.34;3.12;12) | 0.98 (0.67;1.32;84) | U-Test | W=834.5 | **<0.001 (<0.001)** | 0.656 |
| HDL cholesterol (mmol/L) | 1.505 (1.133;1.598;12) | 1.550 (1.255;1.828;84) | U-Test | W=402.0 | 0.261 (0.348) | -0.202 |
| Glucose (mmol/L) | 5.900 (5.580;6.505;12) | 4.935 (4.683;5.215;82) | U-Test | W=944.0 | **<0.001 (<0.001)** | 0.919 |
| sRAGE (pg/mL) | 1386 (970;1928;12) | 1584 (1042;2663;84) | U-Test | W=438.0 | 0.468 (0.535) | -0.131 |
| VEGF (pg/mL) | 193.9 (148.2;251.2;12) | 172.4 (139.1;269.8;84) | U-Test | W=525.0 | 0.820 (0.820) | 0.042 |

*Abbreviations:* BMI=body mass index, CRP=C-reactive protein, FDR=false discover rate-corrected p-value, I-FABP=intestinal fatty acid binding protein, IL-18=interleukin 18, IL-6=interleukin 6, LBP=lipopolysaccharide binding protein, MCP-1=monocyte chemoattractant protein 1, PANSS=positive and negative syndrome scale [PANSS scores were corrected (corr.) by subtraction of minimum scores, which represented no symptoms], HDL=high density lipoprotein, sRAGE=soluble receptor for advanced glycation end products, TNF-a=tumor necrosis factor alpha, VEGF=vascular endothelial growth factor.

# **Supplementary Table 5: Summary of correlation and prediction results for LBP**

| **Predictor Domain** | **Model Pseudo-R²** | **Variable** | **Group** | **Spearman r** | **FDR-corrected p** | **Random Forest Variable Importance** |
| --- | --- | --- | --- | --- | --- | --- |
| Innate immunity-related | 0.354 (All) | CRP | All | 0.614 | <0.001*** | High |
|  | 0.273 (Sz) | CRP | Sz | 0.614 | <0.001*** | High |
|  | 0.449 (Controls) | CRP | Controls | 0.633 | <0.001*** | High |
|  |  | Neutrophils | All | 0.401 | <0.001*** | High |
|  |  | Neutrophils | Sz | 0.394 | <0.001*** | High |
|  |  | Neutrophils | Controls | 0.355 | 0.002** | Low |
|  |  | Monocytes | All | 0.281 | <0.001*** | Low |
|  |  | Monocytes | Sz | 0.316 | 0.004** | Moderate |
|  |  | Monocytes | Controls | 0.149 | 0.272 | Low |
|  |  | IL-18 | All | 0.214 | 0.006** | Moderate |
|  |  | IL-18 | Sz | 0.175 | 0.162 | Moderate |
|  |  | IL-18 | Controls | 0.241 | 0.043* | Low |
| MetS-related | 0.104 (All) | Waist circumference | All | 0.309 | <0.001*** | High |
|  | 0.100 (Sz) | Waist circumference | Sz | 0.315 | 0.017* | High |
|  | 0.048 (Controls) | Waist circumference | Controls | 0.311 | 0.017* | High |
|  |  | Systolic BP | All | 0.220 | 0.009** | Moderate |
|  |  | Systolic BP | Sz | 0.260 | 0.044* | Low |
|  |  | Systolic BP | Controls | 0.079 | 0.511 | Low |
|  |  | Cholesterol | All | 0.164 | 0.034* | Moderate |
|  |  | Cholesterol | Sz | 0.213 | 0.077 | Moderate |
|  |  | Cholesterol | Controls | 0.180 | 0.232 | Low |
| Demographic | 0.097 (All) | BMI | All | 0.304 | <0.001*** | High |
|  | -0.011 (Sz) | BMI | Sz | 0.275 | 0.028* | Moderate |
|  | 0.139 (Controls) | BMI | Controls | 0.369 | <0.001*** | High |
|  |  | Age | All | 0.133 | 0.090 (ns) | Low |
|  |  | Sex | All | ns | 0.429 | Low |
|  |  | Smoking status | All | ↑ in smokers | 0.006** | High |
| Clinical | -0.279 (Sz) | PANSS subscales, illness duration | Sz | ns | ns | Low |

**Annotations:**

- Significance levels: p < 0.05*, < 0.01**, < 0.001***, "ns" = not significant.
- Variable importance in random forest models was interpreted using thresholds for percentage increase in mean squared error (%IncMSE): values <20% were considered low, 20–50% as moderate, and >50% as high importance.
- Random forest pseudo-R² thresholds: <0.20 = negligible, 0.20–0.40 = moderate, >0.40 = strong predictive utility.

# **Supplementary Table 6: Summary of Correlation and Prediction Results for I-FABP**

| **Predictor Domain** | **Model Pseudo-R²** | **Variable** | **Group** | **Spearman r** | **FDR-corrected p** | **Random Forest Variable Importance** |
| --- | --- | --- | --- | --- | --- | --- |
| Innate immune | 0.024 (All) | IL-18 | All | 0.199 | 0.046* | High |
|  | 0.167 (Sz) | IL-18 | Sz | 0.336 | 0.008** | High |
|  | -0.023 (Controls) | IL-18 | Controls | 0.047 | 0.740 | Moderate |
|  |  | Monocytes | All | -0.077 | 0.345 | Moderate |
|  |  | Monocytes | Sz | -0.073 | 0.600 | High |
|  |  | Monocytes | Controls | -0.047 | 0.740 | Low |
|  |  | IL-6 | All | -0.101 | 0.345 | Moderate |
|  |  | IL-6 | Sz | -0.073 | 0.600 | Moderate |
|  |  | IL-6 | Controls | -0.138 | 0.698 | Moderate |
| MetS-related | -0.042 (All) | Cholesterol | All | 0.156 | 0.233 | Moderate |
|  | 0.015 (Sz) | Cholesterol | Sz | 0.141 | 0.477 | Low |
|  | -0.117 (Controls) | Cholesterol | Controls | 0.164 | 0.912 | Moderate |
|  |  | VEGF | Sz | -0.231 | 0.234 | Moderate |
| Demographic | 0.015 (All) | BMI | All | 0.016 | 0.823 | Moderate |
|  | 0.061 (Sz) | BMI | Sz | 0.134 | 0.266 | Moderate |
|  | -0.042 (Controls) | BMI | Controls | -0.133 | 0.399 | High |
|  |  | Age | All | 0.118 | 0.327 | Moderate |
|  |  | Sex | All | ns | 0.218 | Low |
|  |  | Smoking status | All | ns | 0.823 | Low |
| Clinical | -0.132 (Sz) | PANSS subscales, illness duration | Sz | ns | ns | Low |

**Annotations:**

- Significance levels: p< 0.05*, < 0.01**, < 0.001***, "ns" = not significant.
- Variable importance in random forest models was interpreted using thresholds for percentage increase in mean squared error (%IncMSE): values <20% were considered low, 20–50% as moderate, and >50% as high importance.
- Random forest pseudo-R² thresholds: <0.20 = negligible, 0.20–0.40 = moderate, >0.40 = strong predictive utility.

**Supplementary Table 7: Correlation between LBP and I-FABP**

|  | **Plasma LBP.conc. (µg/mL)** |
| --- | --- |
| **Serum I-FABP (pg/mL) All subjects** | r=-0.017, p=0.819 (FDR: 0.819), n=185 |
| **Serum I-FABP (pg/mL) Controls** | r=0.013, p=0.901 (FDR: 0.901), n=93 |
| **Serum I-FABP (pg/mL) all Sz patients** | r=0.018, p=0.866 (FDR: 0.866), n=92 |
| **Serum I-FABP (pg/mL) FESz patients** | r=-0.014, p=0.916 (FDR: 0.916), n=59 |
| **Serum I-FABP (pg/mL) RSz patients** | r=0.038, p=0.833 (FDR: 0.833), n=33 |

*Abbreviations:* FESz= first-episode schizophrenia patients, FDR=false discover rate-corrected p-value, I-FABP=intestinal fatty acid binding protein, LBP=lipopolysaccharide binding protein, RSz= relapsed schizophrenia patients.

**Supplementary Table 8: Comparison of non-smoking Sz patients and non-smoking controls.** Demographic data, clinical assessments, LBP and I-FABP measurements, and immune- and metabolic syndrome-related parameters. Data are presented as median (quartile 1; quartile 3; sample size) or number of cases. Significant p-values are highlighted in bold font.

| **Variables** | **Non-smoking Sz** median (Q1,Q3,n) | **Non-smoking controls** median (Q1,Q3,n) | **Test** | **Test value** | **p-value**  **(FDR)** | **Effect size** (Cliff's delta) |
| --- | --- | --- | --- | --- | --- | --- |
| ***Demographic data & severity of clinical symptoms*** | | | | | | |
| Age (years) | 37.0 (26.8;46.3;42) | 35.5 (27.0;46.0;84) | U-Test | W=1779.0 | 0.940 (0.940) | 0.009 |
| Sex (female/male) | m: 20 / f: 22 | m: 52 / f: 32 | Χ²-Test | X²=1.786 | 0.181 (0.544) | 0.136 |
| BMI (kg/m^2^) | 23.51 (21.02;28.22;42) | 23.65 (21.82;26.56;84) | U-Test | W=1744.0 | 0.920 (0.940) | -0.011 |
| Duration of illness (years) | 0.000 (0.000;2.000;41) | - | - | - | - | - |
| PANSS total corr. (score) | 38.50 (28.00;51.25;42) | - | - | - | - | - |
| PANSS-P corr. (score) | 12.00 (7.00;16.00;42) | - | - | - | - | - |
| PANSS-N corr. (score) | 10.00 (4.00;15.25;42) | - | - | - | - | - |
| PANSS-G corr. (score) | 16.00 (11.75;22.25;42) | - | - | - | - | - |
| ***Leaky gut markers*** | | | | | | |
| Plasma LBP (µg/mL) | 19.35 (14.70;25.42;42) | 17.69 (13.83;23.41;84) | U-Test | W=2006.0 | 0.211 (0.211) | 0.137 |
| Serum I-FABP(pg/mL) | 180.7 (57.9;343.2;40) | 320.3 (174.6;536.5;83) | U-Test | W=1079.0 | **0.002 (0.003)** | -0.350 |
| ***Immune-related parameters*** | | | | | | |
| Neutrophils (×10⁹/L) | 4.45 (3.39;5.97;42) | 2.88 (2.35;3.78;82) | U-Test | W=2734.0 | **<0.001 (<0.001)** | 0.588 |
| Monocytes (×10⁹/L) | 0.53 (0.43;0.70;42) | 0.42 (0.32;0.59;82) | U-Test | W=2307.5 | **0.002 (0.007)** | 0.340 |
| CRP (mg/L) | 0.90 (0.60;2.75;42) | 0.90 (0.50;1.70;83) | U-Test | W=1990.0 | 0.195 (0.380) | 0.142 |
| IL-18 (pg/mL) | 206.2 (169.0;295.8;41) | 204.6 (160.0;267.1;84) | U-Test | W=1853.0 | 0.493 (0.690) | 0.076 |
| IL-6 (pg/mL) | 36.59 (25.09;50.55;35) | 28.92 (17.74;66.81;79) | U-Test | W=1584.0 | 0.217 (0.380) | 0.146 |
| TNF-α (pg/mL) | 16.66 (14.37;28.34;41) | 20.98 (11.27;35.70;81) | U-Test | W=1590.0 | 0.704 (0.807) | -0.042 |
| MCP-1 (pg/mL) | 321.4 (235.6;423.9;41) | 303.6 (225.5;407.0;83) | U-Test | W=1748.0 | 0.807 (0.807) | 0.588 |
| ***Metabolic syndrome-related parameters*** | | | | | | |
| MetS (yes/no) | yes: 6 / no: 36 | yes: 12 / no: 72 | Χ²-Test | X²=0.000 | 1.000 (1.000) | 0.000 |
| Waist circumference (cm) | 89.00 (83.00;103.00;41) | 89.50 (80.00;96.75;84) | U-Test | W=1874.5 | 0.424 (0.636) | 0.089 |
| Systolic blood pressure (mmHg) | 122.5 (110.0;140.0;42) | 120.0 (110.0;129.5;84) | U-Test | W=2127.5 | 0.056 (0.253) | 0.206 |
| Diastolic blood pressure (mmHg) | 80.00 (70.00;90.00;42) | 80.00 (70.00;81.50;84) | U-Test | W=1812.5 | 0.797 (0.896) | 0.027 |
| Triglycerides (mmol/L) | 0.89 (0.66;1.12;42) | 1.015 (0.695;1.427;84) | U-Test | W=1459.0 | 0.115 (0.345) | -0.173 |
| HDL cholesterol (mmol/L) | 1.41 (1.22;1.82;42) | 1.55 (1.29;1.83;84) | U-Test | W=1537.5 | 0.242 (0.545) | -0.128 |
| Glucose (mmol/L) | 5.18 (4.420;5.79;42) | 4.97 (4.70;5.32;82) | U-Test | W=1905.0 | 0.335 (0.603) | 0.106 |
| sRAGE (pg/mL) | 1649 (931;2264;42) | 1582 (1047;2657;84) | U-Test | W=1679.0 | 0.662 (0.851) | -0.048 |
| VEGF (pg/mL) | 240.1 (163.0;309.1;40) | 175.7 (139.9;252.0;84) | U-Test | W=2099.0 | 0.025 (0.228) | 0.249 |

*Abbreviations:* BMI=body mass index, CRP=C-reactive protein, FDR=false discover rate-corrected p-value, I-FABP=intestinal fatty acid binding protein, IL-18=interleukin 18, IL-6=interleukin 6, LBP=lipopolysaccharide binding protein, MCP-1=monocyte chemoattractant protein 1, PANSS=positive and negative syndrome scale [PANSS scores were corrected (corr.) by subtraction of minimum scores, which represented no symptoms], HDL=high density lipoprotein, sRAGE=soluble receptor for advanced glycation end products, TNF-a=tumor necrosis factor alpha, VEGF=vascular endothelial growth factor.

# **Supplementary Table 9: Summary of correlation and prediction results for LBP in *non-smokers***

| **Predictor Domain** | **Model Pseudo-R²** | **Variable** | **Group** | **Spearman r** | **FDR-corrected p** | **Random Forest Variable Importance** |
| --- | --- | --- | --- | --- | --- | --- |
| Innate immune | 0.245 (All) | CRP | All | 0.602 | <0.001*** | High |
|  | 0.347 (Sz) | CRP | Sz | 0.675 | <0.001*** | High |
|  | 0.385 (Controls) | CRP | Controls | 0.537 | <0.001*** | High |
|  |  | Neutrophils | All | 0.360 | <0.001*** | Moderate |
|  |  | Neutrophils | Sz | 0.373 | 0.046* | Low |
|  |  | Neutrophils | Controls | 0.286 | 0.032* | Low |
|  |  | Monocytes | All | 0.242 | 0.016* | Low |
|  |  | Monocytes | Sz | 0.359 | 0.046* | Low |
|  |  | Monocytes | Controls | 0.077 | 0.806 | Low |
| MetS-related | 0.052 (All) | Waist circumference | All | 0.339 | <0.001*** | High |
|  | 0.087 (Sz) | Waist circumference | Sz | 0.448 | 0.027* | High |
|  | 0.026 (Controls) | Waist circumference | Controls | 0.262 | 0.051 | High |
|  |  | Systolic BP | All | 0.309 | 0.002** | Low |
|  |  | Systolic BP | Sz | 0.401 | 0.034* | Low |
|  |  | Systolic BP | Controls | 0.149 | 0.277 | Low |
|  |  | Cholesterol | All | 0.169 | 0.116 | High |
|  |  | Cholesterol | Sz | 0.080 | 0.822 | Low |
|  |  | Cholesterol | Controls | 0.333 | 0.016* | Moderate |

**Annotations:**

- Significance levels: p < 0.05*, < 0.01**, < 0.001***, "ns" = not significant.
- Variable importance in random forest models was interpreted using thresholds for percentage increase in mean squared error (%IncMSE): values <20% were considered low, 20–50% as moderate, and >50% as high importance.
- Random forest pseudo-R² thresholds: <0.20 = negligible, 0.20–0.40 = moderate, >0.40 = strong predictive utility.
